# Barriers to Timely Referral of Children Born with Myelomeningocele in Zambia

**DOI:** 10.3390/jcm14165721

**Published:** 2025-08-13

**Authors:** Rya Muller, Kabelele Sipalo, Caitlyn Beals, Angela Chazura, Stephanie Chola, Roxanna Garcia, Brooks Jackson, Joseph Feinglass, Kirill V. Nourski, Marie-Renee Mala Wa Mpoyi, Humphrey Kunda, Rebecca Reynolds

**Affiliations:** 1 Department of Neurosurgery, Northwestern University Feinberg School of Medicine, Chicago, IL 60611, USA; rya.muller@northwestern.edu (R.M.); roxanna.garcia@northwestern.edu (R.G.); 2 Division of Neurological Surgery, Department of Surgery, University Teaching Hospitals—Adult Hospital, Lusaka 10101, Zambia; kabelele.sipalo27@gmail.com (K.S.); humphrey.kunda@uth.gov.zm (H.K.); 3 Carver College of Medicine, University of Iowa, Iowa City, IA 52242, USA; caitlynbeals21@gmail.com; 4 House of Hope Spina Bifida Hydrocephalus Foundation, Lusaka 10101, Zambia; angelachazura1@gmail.com (A.C.); stephaniemusondachola@gmail.com (S.C.); 5 Department of Pathology, University of Iowa Hospitals and Clinics, Iowa City, IA 52242, USA; brooks-jackson@uiowa.edu; 6 Division of General Internal Medicine, Northwestern University Feinberg School of Medicine, Chicago, IL 60611, USA; j-feinglass@northwestern.edu; 7 Department of Neurosurgery, University of Iowa Hospitals and Clinics, Iowa City, IA 52242, USA; 8 Iowa Neuroscience Institute, University of Iowa, Iowa City, IA 52242, USA; 9 Neonatal Intensive Care Unit, University Teaching Hospitals—Women and Newborn Hospital, Lusaka 10101, Zambia; somwefyfy65@gmail.com

**Keywords:** barriers to care, neural tube defect, LMIC, diagnosis, prenatal care, congenital abnormalities, surgical closure

## Abstract

**Background**: Congenital anomalies impact 52 million infants worldwide with an estimated 94% living in low- and middle-income countries (LMICs). Approximately 200,000 children are born with a neural tube defect (NTD) in LMICs annually. Zambia is an LMIC with a high burden of myelomeningocele (MMC; a severe form of NTD). This study sought to characterize the barriers influencing access to healthcare for children born with MMC in Zambia. **Methods**: Two cross-sectional surveys were administered to healthcare providers at referring public health facilities and mothers of infants born with MMC undergoing surgical closure. The survey among mothers was nested in a longitudinal study evaluating surgical closure in Lusaka, Zambia from 28 May 2024 to 21 January 2025. **Results**: Sixty-nine mother–MMC baby dyads and 123 providers from 21 facilities were enrolled in the study. The median age at presentation for MMC was 7.5 (range 0–244) days old. Most patients were referred from rural district hospitals (51%; *n* = 35) and travelled greater than 250 km to access care (80%; *n* = 55). Seventy-seven percent (*n* = 53) of mothers reported receiving at least one antenatal ultrasound, with 62% (*n* = 43) undergoing an ultrasound after 20 weeks estimated gestational age. Of these, only 3% (*n* = 2) received an MMC diagnosis prior to delivery. Referring patients with MMC for further care greater than six hours after birth was reported by 59% providers (*n* = 73). Hospitals further away from the tertiary center were more likely to report late referrals (*p* < 0.001). **Conclusions**: There is a delay in the diagnosis and referral of infants with MMC to specialized care in Zambia, which may be attributed to inadequate in utero diagnosis capabilities and distance from the tertiary facility. Improving the accuracy of prenatal diagnosis and strengthening referral pathways to facilitate access to care among infants with MMC in Zambia are important for improving incidence and outcomes.

## 1. Introduction

The diagnosis and management of spina bifida varies dramatically around the world. While child mortality has decreased in the last two decades, the global proportion of under-five deaths attributed to congenital abnormalities has increased from 5% in 2000 to 8% in 2022 [1]. In 2021, it was estimated that there were 52 million children with a congenital abnormality, the majority of whom are living in low- and middle-income countries (LMICs) [2]. Neural tube defects (NTDs) are the third most common form of congenital abnormality globally, while in Sub-Saharan Africa they are the second most common [2]. NTDs are congenital malformations of the central nervous system and have been linked to maternal folate deficiencies [3,4]. While several countries have decreased (but not eliminated) the incidence of NTDs through the mandatory fortification of food with folate, the majority of countries in Europe, Asia, and Africa are yet to implement such programs [5].

Myelomeningocele (MMC), a severe type of NTD, results in severe disabilities and high mortality rates; it is estimated that 140 of 1000 patients born with NTDs in Sub-Saharan Africa will die within 5 years [6]. The early surgical closure of MMC, defined as fetal closure or closure within 48 h of birth, is thought to reduce morbidity and mortality [7]. Large variation in the mean age at the time of surgery has been reported from within the region, from 11 days in Ethiopia to 274 days in Sudan [8,9,10,11]. It is estimated that patients in Sub-Saharan Africa face many barriers to care due to limited resources, which delay the time to surgery and contribute to high infant and under-five mortality. The 3DM was developed in 1994 to identify factors that contribute to maternal death [12]. This model identified three phases of delay that affect access to healthcare: (1) delays seeking care, (2) delays reaching care, and (3) delays receiving care. The first delay is associated with cultural and community factors that impact an individual’s decision to seek care. The second delay relates to an individual’s ability to reach care, including geographical/ transportation factors. The third delay is associated with the availability of services offered at the healthcare institution.

Zambia, a Sub-Saharan African country of about 21 million people, has an under-five mortality rate four times that of the United States [6]. There is currently no national data, statistics, or registry available regarding prevalence of MMC in Zambia. However, Zambia has a high anecdotal burden of MMC based upon the experience of the study authors currently practicing medicine in Zambia. Furthermore, patients face many barriers to accessing care; in 2016, a study investigating maternal experience found that Zambian families face several barriers to accessing treatment, including access to transport, access to information, and concerns about the acceptability of medical care [13]. The average age at presentation for surgical MMC treatment in Zambia is nine days, and the average age at surgery is 21 days [14]. This delay is thought to contribute to the high mortality rate for these patients [14]. The surgical closure of MMC in Zambia is primarily offered at one tertiary care hospital in the capital, Lusaka. Most patients initially present at district hospitals or clinics for prenatal care, diagnosis, and initial MMC evaluation. Thereafter they are referred to the tertiary care hospital for specialized care. Delays in accessing adequate prenatal care and diagnosis can contribute to delays in surgical care, as patients diagnosed prenatally can be referred to the tertiary care hospital prior to birth. Additionally, there are a variety of challenges to early referral, including patient or provider education, financial insecurity, transportation costs, and the remote geographical location [15].

The 3DM provides a framework for identifying barriers to care and has been applied beyond maternal death and in LMIC settings [16]. However, no research has been conducted using the 3DM to identify the barriers to care for myelomeningocele in Zambia. This study sought to characterize the diagnosis of MMC at district hospitals in Zambia and identify challenges related to patient transfer to a tertiary center for specialized neurosurgical care. Utilizing the 3DM, we performed a needs assessment through a cross-sectional survey with providers at district hospitals and mother–MMC baby dyads. The primary goal of the study was to identify targets for improving the care of children born with MMC in Zambia, which could have a downstream impact on timing to surgical intervention, complications, and the infant mortality rate.

## 2. Materials and Methods

### 2.1. Sample Selection, Recruitment, and Data Collection

The study cohort consisted of providers at public district hospitals in Zambia and mothers of babies born with spina bifida enrolled in a longitudinal study from 28 May 2024 to 21 January 2025. Written informed consent was obtained from each participant, and the survey was administered by local and international research assistants and translated to the local dialects, as necessary, for the mothers of children born with spina bifida. Surveys were developed by the research team in collaboration with local experts (AC, SC, KS, MRMWA, HK), with 20+ years of experience caring for patients with MMC in Zambia. Surveys underwent several rounds of revisions and expert review to ensure cultural relevancy and clarity. Surveys were not validated due to resource constraints. Surveys were administered by two local research assistants (AC, SC) and two international research assistants (RM, CB).

Healthcare provider participants were recruited from public health facilities through site visits. Sites that historically referred the largest number of patients with MMC were targeted for this study; however, resource limitations affected the number of sites visited during the study. During each site visit, an informational presentation provided by the study team on the importance of the early transfer and treatment of patients with MMC was presented. Inclusion criteria were as follows: (1) healthcare provider employed at a public district hospital who cares for patients with MMC, (2) English speaking without the need for a translator, and (3) ability to provide written informed consent. Providers completed a cross-sectional written survey, comprising 22 multiple choice and three free response questions. All providers included in this study were fluent in English.

In order to capture the clinical journey and demographic profile of patients with MMC, data were collected from mother–baby dyads, as there is no electronic medical record available in the public health system. These surveys were available in English and Nyanja (translated by a local Zambian translator); however, most mothers enrolled in the study did not read or write, so the survey was verbally translated by local research assistants (AC, SC) fluent in English and Nyanja. Research assistants utilized the teach back method with translated questions to ensure that patients understood the questions being asked and the consent form. Mothers of babies born with MMC were recruited as part of a larger prospective study evaluating the outcomes of surgical closure of MMC at a single tertiary care hospital in Lusaka, Zambia from 28 May 2024 to 21 January 2025. Inclusion criteria were as follows: (1) mother of a baby with MMC who resides in Zambia, (2) baby was less than 1 year of age at surgical closure at the tertiary care center. Many of these mothers initially presented to public health facilities prior to arriving to the tertiary care hospital; however, there was no relationship between provider- and maternal-reported data. By collecting data from providers and patients, this study aimed to comprehensively evaluate potential barriers to care for this population.

### 2.2. Study Variables

Healthcare provider surveys captured demographic data including the geographic region, provider job description, and length of career. The following variables were collected: diagnostic and clinical workflow questions included the timing of diagnosis (prenatal vs. postnatal), average age at diagnosis, where patients are referred, timing of referral, barriers to early referral, availability of antibiotics, and whether patients with MMC commonly receive antibiotics at their facility. The primary outcome of early referral was defined as referral within 6 h after the baby is born or received at the provider’s facility. The type of hospital was defined as a Level 3 hospital (specialist or tertiary care hospital), Level 2 hospital (provincial or general hospitals), and Level 1 (district hospitals, health clinics, or health posts). The morbidity and mortality rates of all babies and MMC babies at the providers’ facility were also estimated. Providers were asked to respond to three free-response questions, which asked them to identify targets for improving the care for mothers and babies with MMC. The complete survey can be found in Table S1.

Mother–baby dyad surveys captured patient demographics. Prenatal details were collected from the mother of the patients with MMC at presentation to the tertiary care hospital. Additional variables included the maternal and infant age at presentation, maternal gravidity and parity, maternal education level, income, location of birth, and distance travelled to the tertiary care hospital. Details regarding antenatal care and the clinical course prior to presentation at the tertiary care hospital were also collected. The survey can be found in Table S2.

Study data were collected and managed using REDCap electronic-data-capture tools (https://redcap.icts.uiowa.edu/redcap/, accessed on 1 July 2025) managed by the University of Iowa [17,18]. Prior to data analysis, a comprehensive data-cleaning process was undertaken to ensure the quality and reliability of the dataset. Raw data were initially obtained from REDCap and consisted of 126 records and 42 variables for the provider survey and 69 records and 17 variables for the maternal survey. Data were manually reviewed by a research assistant (RM), and participants with incomplete data were removed (*n* = 3 for provider survey and *n* = 0 maternal survey).

### 2.3. Statistical Analysis

The Shapiro–Wilk Test was utilized to assess the normality of continuous variables, with *p* > 0.05 suggesting significant departure from normality. All continuous variables except for “Gestational age of first prenatal ultrasound” (*p* = 0.767) were normally distributed (*p* < 0.05). However, due to a large number of outliers, medians and ranges are reported for all summary statistics. Outliers were not removed from this dataset as the aim of our study was to assess a wide range of clinical presentations. Categorical variables are presented as frequencies with percentages. Logistic univariate analysis was conducted comparing the association between late referral (>6 h from birth) and clinical/demographic variables. Statistical significance was set a priori at *p* < 0.05. Variables with *p* < 0.25 based on univariate analysis were included in multivariate binary logistic regression models, with late referral as the primary outcome. Statistical analysis was completed with Stata version 18.0 (https://www.stata.com/; College Station, TX, USA). As this was a hypothesis-generating study, no power analysis was undertaken. The sample size was based on logistical feasibility and calculated to provide reasonable power to detect large differences between groups with >25 mother or provider observations. Datasets were analyzed independently.

## 3. Results

### 3.1. Healthcare Providers Survey

A total of 123 providers were surveyed across 21 sites located in four provinces of Zambia: Lusaka, Central, Eastern and Southern provinces. Provider characteristics are shown in Table 1. The majority of providers (59%, *n* = 72) worked in Level 1 hospitals, and a significant proportion were from facilities within 500 km of the tertiary care hospital.

Data regarding provider-reported clinical and referral workflow are shown in Table 2. Regarding the timing of diagnosis, 53% (*n* = 65) of providers reported diagnosing the patient after birth and 29% (*n* = 35) diagnosing patients on ultrasound prior to birth. The median age of diagnosis was one (0–24) day old. Providers most frequently referred patients directly to a tertiary care hospital for further management (74%, *n* = 91). Providers most frequently referred patients after 6 h of birth (59%, *n* = 73). Most providers experienced no barriers to early referral (95%, *n* = 114). Regarding antibiotics, 98% (*n* = 121) reported that antibiotics were available at their hospital and 82% (*n* = 101) frequently administered antibiotics to patients with MMC.

**Figure 1:**
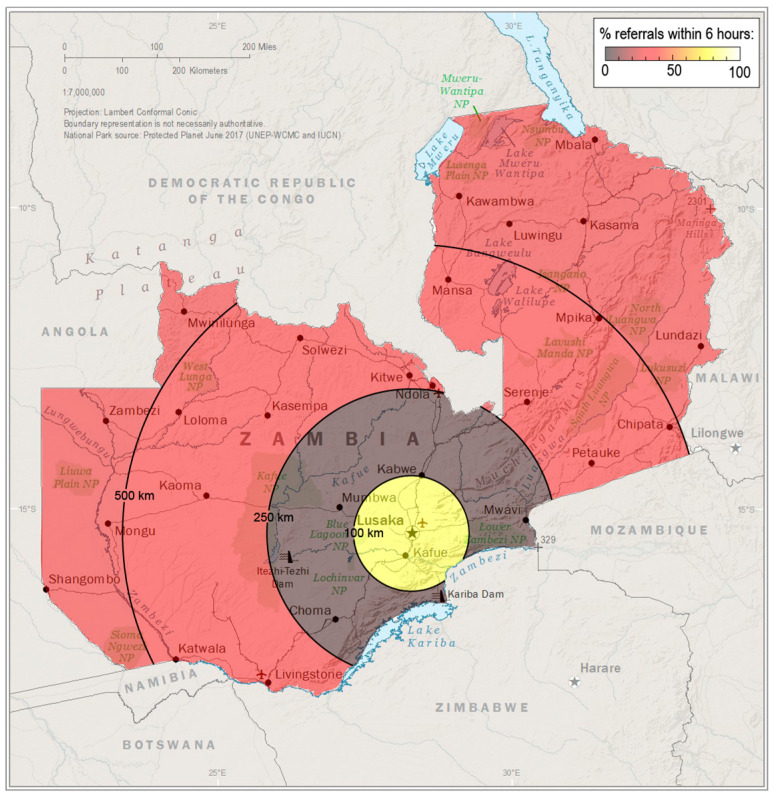
Greater distance from the tertiary care center in Lusaka (star) was associated with a lower odds of early patient referral. Data source: The World Factbook 2024. Washington, DC: Central Intelligence Agency, 2024.

Findings of the logistic univariate and multivariable analyses are shown in Table 3 to identify factors associated with the likelihood of early referral to tertiary care. Providers who worked at hospitals 100–249 km from the tertiary care hospital were 4.30 times more likely to be referred after 6 h compared to those at hospitals <100 km from the tertiary care hospital (*p* < 0.001). Greater distance from the tertiary care center was associated with a lower odds of early referral (Figure 1) after controlling for confounders in the multivariable analysis. The type of provider, work setting, years of experience, antenatal diagnosis, and administering antibiotics to patients with MMC were not statistically significant.

Most providers reported that MMC care for patients in Zambia needs to be improved (94%, *n* = 116). When asked about the factors contributing to the high rates of MMC in Zambia, providers cited maternal nutritional/folic acid deficiency (62%, *n* = 76), maternal delays in or barriers to seeking antenatal care (21%, *n* = 26), a lack of prenatal diagnosis (18%, *n* = 22), environmental exposure (6%, *n* = 7), and the young average maternal age (2%, *n* = 3). Responses regarding targets for improvement of care included providing early referral and repair for patients (75%, *n* = 61), community sensitization and education about MMC (70%, *n* = 57), social and emotional support for mothers of babies with MMC (36%, *n* = 44), improved antenatal care and diagnosis (33%, *n* = 41), improved healthcare infrastructure (21%, *n* = 26), financial support for mothers (20%, *n* = 25), and early folic acid supplementation (17%, *n* = 21).

### 3.2. Mother–Baby Dyad Survey

A total of 69 mother–baby dyads were surveyed (Table 4). The median age at presentation for the patients with MMC was 7.5 days (IQR 1-19). Most patients with MMC were born at Level 1 hospitals (90%, *n* = 62). One patient was born at home. Mothers reported travelling far distances to access care, with 80% (*n* = 55) traveling more than 250 km to reach the tertiary care hospital. Regarding antenatal care, 77% (*n* = 53) of mothers reported undergoing at least one antenatal ultrasound, with a median gestational age at first ultrasound of five (IQR 3-7) months. Sixty-two percent (*n* = 43) of patients underwent an anatomy ultrasound after 20 weeks gestational age; however, only 3% (*n* = 2) of patients received an MMC diagnosis prior to birth. Most mothers reported taking folic acid supplements during pregnancy (97%, *n* = 67), with 3% (*n* = 2) taking supplements prior to conception.

## 4. Discussion

There are opportunities to improve care for MMC worldwide and Zambia is no exception. The crux of adequate MMC management rests upon timely diagnosis and referral. While 62% of mothers in this study sought prenatal care and received at least one prenatal ultrasound, fewer than 5% received an antenatal MMC diagnosis. Furthermore, fewer than half of infants were referred for a surgical evaluation immediately after birth. As is common for rural healthcare globally, Zambian patients located in the rural communities that were further from the tertiary care hospital faced greater delays in receiving a referral (*p* < 0.001, Figure 1). These findings identify the primary reasons for the delayed initiation of hospital transfer to the hospital, specifically delays in accessing and receiving care, and highlight areas for the improvement of care for MMC and potentially other patients with congenital malformations.

### 4.1. Limitations on Access to Care

Improving access to surgical care for MMCs and other congenital malformations is vital for decreasing infant and child mortality. In 2019, 550,000 children died from a congenital malformation, the majority of which were in LMICs [19]. In high-income countries, delayed surgical closure, defined by North American standards as greater than 48 h after birth, has been associated with increased infection rates [20]. In this study, MMC patients presented late for care with an average age of 7.5 days, despite mothers seeking prenatal care and receiving an ultrasound in the second trimester. Several factors contribute to barriers to care in Zambia: lack of prenatal diagnosis, poverty, limited transportation infrastructure, high rates of child marriage, frequent home births, and stigma related to congenital defects [15,21]. Stigma can discourage mothers from seeking care for their babies as they often need to ask their local communities to fundraise for hospital transportation [15,21,22]. The travel distance is substantial after delivery in Zambia, with 80% of patients reported travelling greater than 250 km to access a neurosurgeon. A lack of access to pediatric surgical care is not unique to LMICs. In 2010, there were nearly 12 million children in the United States and Canada that lived greater than 100 km away from pediatric surgical care [23,24]. Telemedicine has been successful in addressing barriers to prenatal and antenatal care for high risk patients in high-income countries [25,26] and improving access to surgical care in LMICs [27]. However, there are several barriers to implementing telemedicine in LMIC settings, including the lack of reliable access to electricity, internet, and electronic devices. Further research is needed to understand the barriers prevalent in these settings and devise culturally acceptable measures to address them.

### 4.2. A Need to Improve Prenatal Ultrasound Diagnostic Capabilities

Second trimester ultrasound can be 90–98% sensitive in detecting spina bifida at a 20-week estimated gestational age for an anatomy ultrasound [28]; however, it is only 4% sensitive in Zambia. In this study, 3% of all mothers received a prenatal diagnosis, which represents a significant barrier to receiving adequate care. This is similar to other studies published in LMICs where antenatal detection rates range from 0 to 19% [29,30,31,32,33]. Low prenatal detection levels could be attributed to several factors. In some settings mothers may not have access to imaging facilities or have poor adherence to screening. While more than half of the mothers obtained an antenatal ultrasound after the second trimester, patients presented late for their first ultrasound, with a median gestational age of five months. Regardless, only two received a prenatal diagnosis. This calls into question whether providers are adequately trained in the radiographic detection of congenital anomalies or have the resources available to detect them. Furthermore, there was no association between the provider type or work experience and early referral, which may suggest the need for improved education across all levels of training. Several initiatives have been implemented to address the underdiagnosis of congenital abnormalities in LMICs, including maternal blood testing, midwife training, and sonographer training programs [34,35,36,37,38]. However, antenatal detection does not independently improve maternal and fetal outcomes. A randomized control trial conducted in several LMICs in Africa demonstrated that despite a 71% rate of subspecialty clinic attendance for mothers with a congenital abnormality detected on ultrasound, prenatal ultrasound did not improve fetal and neonatal mortality [39]. In this study, per provider data, there was no significant association between patients receiving an antenatal diagnosis and early referral. This may be attributed to the recall bias of providers, as many more reported patients receiving antenatal diagnoses than was reported by patients themselves. However, this may also suggest that while an antenatal diagnosis of spina bifida has the potential to increase early referral and maternal education about the disease, it is only as strong as the surrounding healthcare system. Studies and interventions focused on strengthening the healthcare infrastructure in these settings are needed to ensure that patients have access to resources should they receive a prenatal diagnosis.

### 4.3. Prevention of Spina Bifida

NTDs and MMC are considered preventable diseases, through folic acid fortification, but other studies suggest additional risk factors and etiologies. Folic acid fortification prevents 28% of cases of NTDs in the US [40], and the majority of women in this Zambian study also reported taking folic acid during pregnancy. However, timing is the problem; only 3% (*n* = 2) took folic acid prior to conception, which is important since the neural tube defect causing MMC occurs during the first month of pregnancy. There may be several reasons why mothers are not taking folic acid prior to conception, including limited access to healthcare resources, unplanned conception, or a young maternal age [21,41]. The majority of LMICs do not currently have folate fortification programs and face several barriers to developing such programs [5,42]. South Africa enacted the mandatory fortification of bread flour and maize meal in 2003, resulting in a 30.5% decrease in NTDs between 2004 and 2005 [43]. The Nigerian government recently adopted a multi-fortified bouillon cube plan. Bouillon cubes are a frequently included ingredient in West African cooking, and this plan is estimated to save over 57,000 children under 5 in Nigeria between 2023 and 2030 [44,45,46]. These programs could be used as models to enact folate fortification in other LMIC countries, including Zambia.

Genetics, medication exposures, and environmental exposures are also thought to play a role in NTD development [47,48,49]. Recently, high levels of maternal polycyclic aromatic hydrocarbons, which are released during the combustion of fossil fuels, have been associated with increased risks for NTDs [47,49,50,51,52,53,54,55]. In Zambia, as well as many other LMICs, where charcoal remains a common source of indoor heating and cooking, this compound may be implicated. Furthermore, in this study, several providers from Eastern province, where it is estimated that many babies with MMC are born in Zambia, expressed concerns over the impact of the mining explosive used in the region. While the mandatory folate fortification of food is vital for preventing some NTDs, the likely multifactorial etiology of NTDs warrants further investigation to ultimately prevent this disease.

Lastly, several providers indicated that community education and sensitization about MMCs are needed in Zambia. The maternal understanding of the link between folic acid and MMCs in Zambia is low, and local community educational programs have been effective in addressing this problem [13]. Beyond prevention, providers in this study indicated that mothers had limited knowledge on the importance of seeking surgical care for their babies with MMC. This limitation, combined with the stigma that mothers of babies with congenital abnormalities face in Zambian communities, contributes to the delayed time to surgery [15]. However, further research is needed in Zambia and other LMICs to understand the optimal targets for educational programs and cultural context within which they would be delivered.

### 4.4. Policy Implications and Future Directions

Reducing the burden of NTDs is a global priority, corresponding to the fourth goal of the United Nations (UN) Millennium Development campaign to reduce child mortality, as well as the 142nd Session of the World Health Assembly resolution on maternal prenatal nutrition [56,57]. Furthermore, the recent passing of the “Accelerating efforts for preventing micronutrient deficiencies and their consequences, including spina bifida and other neural tube defects, through safe and effective food fortification.” [EB152/CONF./5] at the May 2023 World Health Assembly highlighted the global push to improve efforts towards folate fortification [58]. The potential effectiveness of folate fortification in Zambia has been highlighted in many studies [13,59,60], with one paper calculating that fortifying maize flour in Zambia would save 17,286 disability-adjusted life years (DALYs) per year [59]. Therefore, mandatory folic acid fortification policies in Zambia, specifically of maize, have the potential to significantly reduce the burden of MMC in the country. Local and global stakeholders must work towards creating and passing policy to this effect.

Additionally, the importance of reducing infant and child mortality was globally defined in 2015 as part of the Sustainable Development Goals (SDG), with the UN pledging to reduce neonatal mortality to 12 deaths per 1000 live births and under-five mortality 25 deaths per 1000 live births by 2030 [61]. Part of this commitment includes access to effective prenatal care [61]. As this study emphasized, there is a lack of effective prenatal care, as most women with babies with MMC did not receive an antenatal diagnosis, despite undergoing several antenatal ultrasounds. Our group is already conducting research to evaluate the state of antenatal radiological care in Zambia. However, training is needed to ensure that all individuals conducting or interpreting antenatal scans can detect and diagnose congenital malformations in utero.

### 4.5. Limitations

There are several limitations to this study. Information collected directly from patients was conducted at a single hospital in one country. While this tertiary care hospital serves the majority of Zambia, and therefore the results are mostly likely applicable to Zambians, they are not necessarily applicable to countries outside of Zambia. Additionally, providers attended a workshop about the importance of early referral prior to filling out the survey, which may have introduced response bias. The results may also be subject to recall bias, as providers were asked to recall and estimate information from the previous year. Providers were chosen as part of a convenience sample; not every province in Zambia was represented in this study. Therefore, this may affect the accuracy of the results, and they may not be representative of what all Zambians experience when seeking healthcare for patients with MMC. Additionally, there may be other barriers or information regarding patients with spina bifida that was not reported in the results. The time from referral to arrival at the tertiary care hospital was not collected. This study focused on one aspect of the healthcare system in Zambia; further investigation is needed to characterize the many aspects that contribute to patient presentation, access, and receiving of care. Further, healthcare information is recorded on paper charts in Zambia, which are carried by the patient. These charts have variable amounts of information or levels of detail and are sometimes lost, potentially impacting the accuracy of the results. While patient surveys were translated in Nyanja, they were not back-translated due to resource constraints, which may impact the validity of the questions. Most mothers were illiterate, so the surveys were verbally translated by research assistants. While these research assistants have extensive experience in caring for MMC patients, they are not formally trained in medical translation, which may impact the accuracy of the translated questions and therefore the results. Additionally, all surveys were multiple choice or short response; therefore the absence of Likert scales, vague items, or translation ambiguity may impact the reliability and accuracy of the results. Surveys were also not validated, impacting the external validity of the results. Patients’ parents were also asked to recall information when it was not available in the chart, which may result in recall bias. This is especially relevant when considering the self-reported diagnosis of MMC prior to delivery. While patients may not have been aware of the diagnosis, this does not necessarily mean that the clinical team did not make the diagnosis and either did not communicate the results clearly or chose not to communicate the results at all. Additionally, the two datasets were analyzed independently without linked analysis, which may introduce bias or limit the accuracy of the results. The author team assimilated as much information as was accessible, and as such, despite these limitations, this study offers meaningful insight into the prenatal evaluation of infants with spina bifida in Zambia.

## 5. Conclusions

This is the first study conducted on barriers to referral and early access to surgery for children with MMC in Zambia. Patients with MMC experience significant delays in the referral and evaluation by neurosurgeons in Zambia. These delays are partially linked to distance from the tertiary care hospital and the lack of a prenatal diagnosis of MMC, despite most receiving prenatal ultrasound after 20 weeks gestation. The findings from this study are geographically specific to Zambia and may need adaptation before generalizing to other LMIC settings. However, interdisciplinary collaboration with local providers is vital, and future studies should investigate methods to improve the early diagnosis and timely referral of children born with MMC, as well as other NTDs, in Zambia.

## Figures and Tables

**Table 1:** Provider characteristics surveyed across 21 public health facilities in Zambia (*n* = 123).

Variables	*n* (%)
Type of provider	
Doctor	36 (29)
Nurse	43 (35)
Midwife	34 (28)
Other	10 (8)
Work setting	
Level 3 hospital	19 (15)
Level 2 hospital	22 (18)
Level 1 hospital	72 (59)
Other	10 (8)
Distance from tertiary care center (km)	
0–99	42 (34)
100–249	21 (17)
250–499	49 (40)
500+	11 (9)
Years of experience	
<1 year	9 (7)
1–5 years	45 (37)
6–10 years	35 (29)
11–20 years	25 (20)
>20 years	9 (7)

**Table 2:** Provider-reported referral and clinical patterns (*n* = 123).

Variables	*n* (%)
MMC patients per month, median (range)	1 (0–12)
Diagnosis of MMC	
Before the baby is born (on ultrasound)	35 (29)
I diagnose it after the baby is born	65 (53)
Another provider on my team diagnoses it	10 (8)
Another provider outside my hospital diagnosis it	9 (7)
Other	4 (3)
Age at diagnosis (days), median (range)	1 (0–24)
Where is patient referred?	
Level 3 hospital	91 (74)
Level 2 hospital	10 (8)
Level 1 hospital	3 (3)
Other	19 (15)
When is the baby referred for further management?	
Within 6 h after baby is born or received	50 (41)
7–12 h after baby is born or received	13 (11)
13–24 h after baby is born or received	20 (16)
2–3 days after baby is born or received	19 (15)
More than 3 days after baby is born or received	14 (11)
Unknown	7 (6)
Are babies referred directly to UTH?	
Yes	90 (73)
No	33 (27)
Are babies referred to UTH within the first 3 days of life?	
Yes	79 (90)
No	9 (10)
Barriers to early referral	
No money for transport	0 (0)
No ambulance available for transport	0 (0)
Diagnosis of myelomeningocele not made that quickly	3 (2)
Mother’s preference	0 (0)
Provider’s preference	0 (0)
Other	6 (5)
No barriers reported	114 (95)
Antibiotics available?	
Yes	121 (98)
No	2 (2)
Do MMC patients receive antibiotics	
Yes	101 (82)
No	7 (6)
I do not know	15 (12)

**Table 3:** Univariate and logistic regression analysis for timing to referral (*n* = 123).

Variables	Time to Referral		Univariate		Multivariate	
	≤6 h (*n* = 51) *n* (%)	>6 h (*n* = 72) *n* (%)	Odds Ratio (95% CI)	*p*-Value	Odds Ratio (95% CI)	*p*-Value
Provider				0.427		
Doctor	13 (36)	23 (64)	Ref			
Nurse	17 (40)	26 (60)	−0.15 (−1.06–0.77)	0.159		
Midwife	18 (53)	16 (47)	−0.69 (−1.65–0.27)	0.755		
Other	3 (30)	7 (70)	0.28 (−1.24–1.79)	0.720		
Work setting						
Level 3 hospital	11 (58)	8 (42)	Ref		Ref	
Level 2 hospital	29 (40)	43 (60)	0.69 (−0.46–1.64)	0.273	0.35 (−0.98–1.68)	0.608
Level 1 hospital	6 (27)	16 (73)	−0.71 (−1.74–0.31)	0.173	−0.77 (−2.02–0.49)	0.230
Other	5 (50)	5 (50)	−0.39 (−1.72–0.93)	0.560	−0.98 (−2.54–0.58)	0.216
Distance (km)						
0–99	33 (79)	9 (21)	Ref		Ref	
100–249	1 (5)	20 (95)	4.30 (2.16–6.43)	**<0.001**	4.23 (2.07–6.38)	**<0.001**
250–499	14 (29)	35 (71)	2.22 (1.25–3.18)	**<0.001**	2.41 (1.38–3.44)	**<0.001**
500+	3 (27)	8 (73)	2.28 (0.76–3.80)	**0.001**	1.99 (0.39–3.58)	**0.015**
Experience (yr)						
<1 year	3 (33)	6 (67)	Ref			
1–5 years	18 (40)	27 (60)	0.35 (−0.68–1.38)	0.508		
6–10 years	19 (54)	16 (45)	0.29 (−1.22–1.80)	0.709		
11–20 years	8 (32)	17 (68)	−0.58 (−1.47–0.32)	0.205		
>20 years	3 (33)	6 (67)	0.28 (−1.22–1.80)	0.709		
Antenatal diagnosis	14 (40)	21 (60)	0.08 (−0.71–0.88)	0.835		
Receive antibiotics	43 (43)	58 (58)	−0.26 (−1.21–0.69)	0.593		

**Table 4:** Clinical and maternal data for patients with MMC in Zambia (*n* = 69).

Variables	*n* (%)
Maternal age (years), median (range)	25 (15–43)
Gravidity, median (range)	2 (1–8)
Parity, median (range)	2 (1–8)
Age at presentation (days), median (range)	7.5 (0–244)
Location of birth	
Level 3 hospital	3 (4)
Level 2 hospital	0 (0)
Level 1 hospital	62 (90)
Home	1 (2)
Other	3 (4)
Distance travelled to tertiary care center (km)	
0–99	4 (6)
100–249	10 (14)
250–499	29 (42)
500+	26 (38)
Maternal education	
College or beyond	7 (10)
Secondary school	14 (20)
Primary school	40 (58)
No school	8 (12)
Monthly family income (dollars), median (range)	7.19 (0.04–179.69)
Antibiotics prior to presentation at UTH	40 (58)
Prenatal ultrasound	53 (77)
Gestational age of first prenatal ultrasound (months), median (range)	5 (1–9)
Prenatal ultrasound after 20 weeks gestational age	43 (62)
Prenatal diagnosis of spina bifida	2 (3)
Folic acid intake	
Prior to conception	2 (3)
During pregnancy	67 (97)

## Data Availability

The datasets used and/or analyzed during the current study are available from the corresponding author on reasonable request.

## Supplementary Materials

The following supporting information can be downloaded at: https://www.mdpi.com/article/10.3390/jcm14165721/s1, Table S1: Provider survey; Table S2: Patient survey.

## Abbreviations

The following abbreviations are used in this manuscript:

DALY	disability-life adjusted year
IQR	interquartile range
LMIC	low-middle income country
MMC	myelomeningocele
NTD	neural tube defect
SDG	Sustainable Development Goals
UN	United Nations
