# Supplementary material for: Barriers to Timely Referral of Children Born with Myelomeningocele in Zambia
Source: J Clin Med. 2025 Aug 13;14(16):5721. doi: 10.3390/jcm14165721 (PMC12386297; doi:10.3390/jcm14165721)
Supplement: Supplementary file 1 [file jcm-14-05721-s001.zip › jcm-3760133-supplementary.pdf]

Supplementary Table S1. Provider Survey

Question	Responses
1. Province in Zambia where you currently work?	a. Northwestern<br>b. Western<br>c. Central<br>d. Lusaka<br>e. Southern<br>f. Luapula<br>g. Northern<br>h. Eastern<br>i. Prefer not to answer
2. Where do you primarily work?	a. University Teaching Hospital (Level 3)<br>b. District Hospital (Level 1)<br>c. Clinic (Level 1)<br>d. Other (please describe)
3. What is your primary job description?	a. Doctor<br>b. Midwife<br>c. Nurse<br>d. Other (please describe)
4. How long have you been working in your profession?	a. Less than 1 year<br>b. 1-5 years<br>c. 6-10 years<br>d. 11-20 years<br>e. More than 20 years
5. Where are the greatest number of babies born that you provide care for?	a. Your hospital<br>b. Your clinic<br>c. Another hospital<br>d. Another clinic<br>e. Patient’s home<br>f. Other (please describe)
6. Do you know what is a myelomeningocele (spina bifida)?	a. Yes<br>b. No ⇒ If no is answered, then discontinue the survey.
7. Do you know how to diagnose or recognize myelomeningocele in an infant?	a. Yes<br>b. No
8. How many times per month do you take care of a newborn infant with myelomeningocele?	a. (Indicate # of babies monthly)
9. When do the greatest number of your patients receive a diagnosis of myelomeningocele?	a. Before the baby is born (on ultrasound)<br>b. I diagnose it after the baby is born<br>c. Another provider on my team diagnoses it<br>d. Another provider outside my hospital or clinic diagnoses it<br>e. Other (please describe)
10. When a baby is diagnosed with myelomeningocele, typically how old is the baby?	a. (Indicate age in days)
11. When a baby is diagnosed with myelomeningocele at your facility, where do you most commonly refer the patient for further evaluation and management?	a. Next level clinic<br>b. District hospital<br>c. General hospital<br>d. University Teaching hospital (UTH or Levy)<br>e. Other (please describe)
12. When a baby is diagnosed with myelomeningocele, when do you most commonly refer the patient for further evaluation and management?	a. Within 6 hours after baby is born or received<br>b. 7-12 hours after baby is born or received<br>c. 13-24 hours after baby is born or received<br>d. 2 days after baby is born or received<br>e. 3 days after baby is born or received<br>f. Later than 3 days (please specify)
13. Do you ever refer/ transfer babies with myelomeningocele (born or received) at your facility directly to the UTH or Levy?	a. Yes<br>b. No
14. If answer to 13 is YES, do you ever refer/ transfer babies with myelomeningocele (born or received) at your facility to the UTH or Levy within the first 3 days of life?	a. Yes<br>b. No
15. If answer to 14 is NO, give reasons. (check all that apply)	a. No money for transport<br>b. No ambulance available for transport<br>c. Diagnosis of myelomeningocele not made that quickly<br>d. Mother’s preference<br>e. I don’t think it’s necessary<br>f. Other (please describe)
16. Does your facility have antibiotics available to give to patients?	a. Yes<br>b. No<br>c. I don’t know

17. If answer is YES to 16, does the patient commonly receive antibiotics at your facility after they are born with myelomeningocele?	a. Yes (if yes, which antibiotics typically are administered and for how long)<br>b. No<br>c. I don't know
18. Approximately, what percentage of all infants born at your facility would you estimate develop fevers or hypothermia?	a. (Indicate a percentage out of 100)
19. Approximately, what percentage of all infants born at your facility would you estimate die?	a. (Indicate a percentage out of 100)
20. Approximately, what percentage of infants born with myelomeningocele at your facility would you estimate develop fevers or hypothermia?	a. (Indicate a percentage out of 100)
21. Approximately, what percentage of infants born with myelomeningocele at your facility would you estimate die?	a. (Indicate a percentage out of 100)
22. Do you think that early surgical repair for myelomeningocele will help babies born with this condition survive?	a. Yes<br>b. No<br>c. I don't know
23. Zambia has a high incidence of newborns born with myelomeningocele. From your perspective, why do you think this is the case?	Free text response
24. How do you think the care of infants born in Zambia with myelomeningocele could be improved?	Free text response
25. How do you think the care of mothers with an infant born in Zambia with myelomeningocele could be improved?	Free text response
26. Are you interested in learning more about how to improve outcomes for infants born with myelomeningocele in Zambia?	a. Yes<br>b. No

Supplementary Table S2. Patient Survey

Question	Responses
1. What is the age of the mother?	
2. What is the highest school education that the mother received?	a. No school<br>b. Primary school<br>c. Secondary school<br>d. College or beyond
3. Did the mother receive prenatal care? (defined as at least one prenatal healthcare visit)	a. Yes<br>b. No
4. In what month of pregnancy was the first ultrasound?	
5. Did the mother have more than one ultrasound during pregnancy?	a. Yes<br>b. No
6. In what month of pregnancy was the last ultrasound?	
7. Did any ultrasound show a baby with spina bifida?	a. Yes<br>b. No
8. Did the mother know that she was going to have a baby born with spina bifida?	a. Yes<br>b. No
9. Did the mother take folic acid vitamin prior to getting pregnant?	a. Yes<br>b. No
10. Did the mother take folic acid vitamin while pregnant?	a. Yes<br>b. No
11. How many times has the mother been pregnant?	
12. How many living children does the mother have?	
13. In what location was the patient born?	a. Home<br>b. Clinic<br>c. District Hospital<br>d. UTH<br>e. Other (please list)
14. Where was the patient born?	a. Northwestern Province<br>b. Western Province<br>c. Central Province<br>d. Lusaka Province<br>e. Southern Province<br>f. Laupula Province<br>g. Northern Province<br>h. Eastern Province<br>i. Copperbelt Province<br>j. Muchinga Province<br>k. Other (please list)
15. Age at presentation to UTH	
16. What is the average monthly household income (in Kwacha)?	
17. Was the patient on antibiotics prior to first neurosurgical evaluation?	a. Yes<br>b. No
